# Chemical fingerprinting of single glandular trichomes of *Cannabis sativa* by Coherent anti-Stokes Raman scattering (CARS) microscopy

**DOI:** 10.1186/s12870-018-1481-4

**Published:** 2018-11-12

**Authors:** Paul Ebersbach, Felix Stehle, Oliver Kayser, Erik Freier

**Affiliations:** 1 Leibniz-Institut für Analytische Wissenschaften – ISAS – e.V, 44227 Dortmund, Germany; 2 TU Dortmund, Technische Biochemie, 44227 Dortmund, Germany

**Keywords:** Coherent anti-stokes Raman scattering, *Cannabis sativa*, Trichomes, Secondary metabolites, Hyperspectral imaging, Unmixing, Two-photon fluorescence, Mapping, THCA, CBDA

## Abstract

**Background:**

*Cannabis* possesses a rich spectrum of phytochemicals i.e. cannabinoids, terpenes and phenolic compounds of industrial and medicinal interests. Most of these high-value plant products are synthesised in the disk cells and stored in the secretory cavity in glandular trichomes. Conventional trichome analysis was so far based on optical microscopy, electron microscopy or extraction based methods that are either limited to spatial or chemical information. Here we combine both information to obtain the spatial distribution of distinct secondary metabolites on a single-trichome level by applying Coherent anti-Stokes Raman scattering (CARS), a microspectroscopic technique, to trichomes derived from sepals of a drug- and a fibre-type.

**Results:**

Hyperspectral CARS imaging in combination with a nonlinear unmixing method allows to identify and localise Δ^9^-tetrahydrocannabinolic acid (THCA) in the secretory cavity of drug-type trichomes and cannabidiolic acid (CBDA)/myrcene in the secretory cavity of fibre-type trichomes, thus enabling an easy discrimination between high-THCA and high-CBDA producers. A unique spectral fingerprint is found in the disk cells of drug-type trichomes, which is most similar to cannabigerolic acid (CBGA) and is not found in fibre-type trichomes. Furthermore, we differentiate between different cell types by a combination of CARS with simultaneously acquired two-photon fluorescence (TPF) of chlorophyll *a* from chloroplasts and organic fluorescence mainly arising from cell walls enabling 3D visualisation of the essential oil distribution and cellular structures.

**Conclusion:**

Here we demonstrate a label-free and non-destructive method to analyse the distribution of secondary metabolites and distinguish between different cell and chemo-types with high spatial resolution on a single trichome. The record of chemical fingerprints of single trichomes offers the possibility to optimise growth conditions as well as guarantee a direct process control for industrially cultivated medicinal *Cannabis* plants. Moreover, this method is not limited to *Cannabis* related issues but can be widely implemented for optimising and monitoring all kinds of natural or biotechnological production processes with simultaneous spatial and chemical information.

**Electronic supplementary material:**

The online version of this article (10.1186/s12870-018-1481-4) contains supplementary material, which is available to authorized users.

## Background

*Cannabis*, the most widely used illicit drug worldwide [1], experiences a renaissance in medical use since the discovery of the endocannabinoid system [2]. For example, tetrahydrocannabinol (THC) is used for the treatment of the symptoms of e.g. neurological diseases [3], multiple sclerosis [4] or cancer [5]. Other cannabinoids like cannabidiol (CBD), terpenes and phenolic compounds show further pharmacological effects, which make this plant a highly interesting pharmaceutical target [6]. The biosynthesis of these metabolites typically occurs in specialised plant surface structures, so-called glandular trichomes. Trichomes are epidermal structures that are widespread among plants showing a multitude of functions in physical as well as biological stress responses or ecological interactions [7–9] and can be divided into non-glandular and glandular trichomes. *Cannabis* exhibits both, two different types of non-glandular hairs and two groups of glandular trichomes [10]. The first group of glands is a collection of trichomes with different shapes and architecture but they all have a small swollen head on a short stalk. The second group are capitate glands that have a large globular head and (massive) stalks. They are produced on flowering bracts of female flowers and on anthers of male flowers. These glands are thought to be the primary site of cannabinoid biosynthesis and storage [10, 11].Therefore, only glandular trichomes were analysed in this study.

**Fig. 1:**
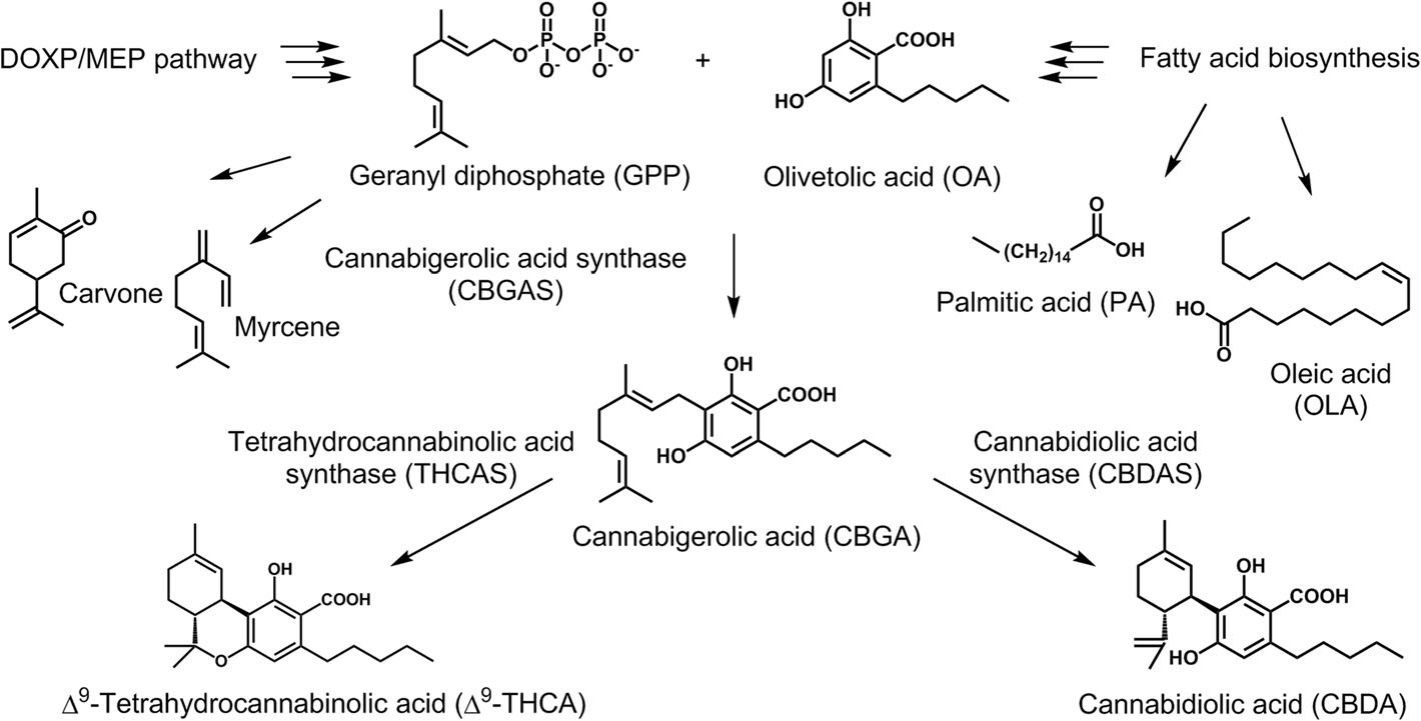
Biochemical pathway of cannabinoid synthesis in *C. sativa.* CBGA, the central intermediate of the cannabinoid pathway, is formed from GPP and OA. Subsequently CBGA is further converted to the acidic forms of THC and CBD by two different oxidoreductases THCAS and CBDAS. Additionally, highly abundant monoterpenes and fatty acids from the essential oil of glandular trichomes are shown [42, 43]

Previous studies have shown that within glandular trichomes the synthesis of diverse metabolites occurs in disk cells whereas the accumulation is facilitated in the adjacent secretory cavity [12]. Cannabigerolic acid (CBGA), the central precursor of the cannabinoids, is formed from geranyl diphosphate (GPP) and olivetolic acid (OA), derived from the DOXP/MEP and polyketide pathway, respectively (Fig. 1) [13]. Starting from CBGA numerous cannabinoids are synthesised with ∆^9^-tetrahydrocannabinolic acid (THCA) and cannabidiolic acid (CBDA) as the most abundant ones [13–17]. According to the THCA and CBDA content *Cannabis* plants are classified as drug-type (THCA-rich, CBDA-poor), intermediate (THCA-medium / -poor, CBDA-rich), and fibre-type (THCA-poor, CBDA-rich) plants [13, 18].

Recently, the analysis of gland-derived expressed sequence tags followed by quantitative polymerase chain reaction analysis showed that almost all candidate genes of the cannabinoid pathway are preferentially expressed in glandular trichomes [19]. The determination of the essential oil composition so far has been performed by GC/MS [20], LC/MS [21, 22] or NMR analysis [22], or the concentration of THCA was estimated by fluoroimmunoassays of *Cannabis* extracts [23]. Due to the destructive nature of these techniques and the large amount of glands needed, information on single trichomes or spatial localisation of the metabolites within a trichome are lost.

**Fig. 2:**
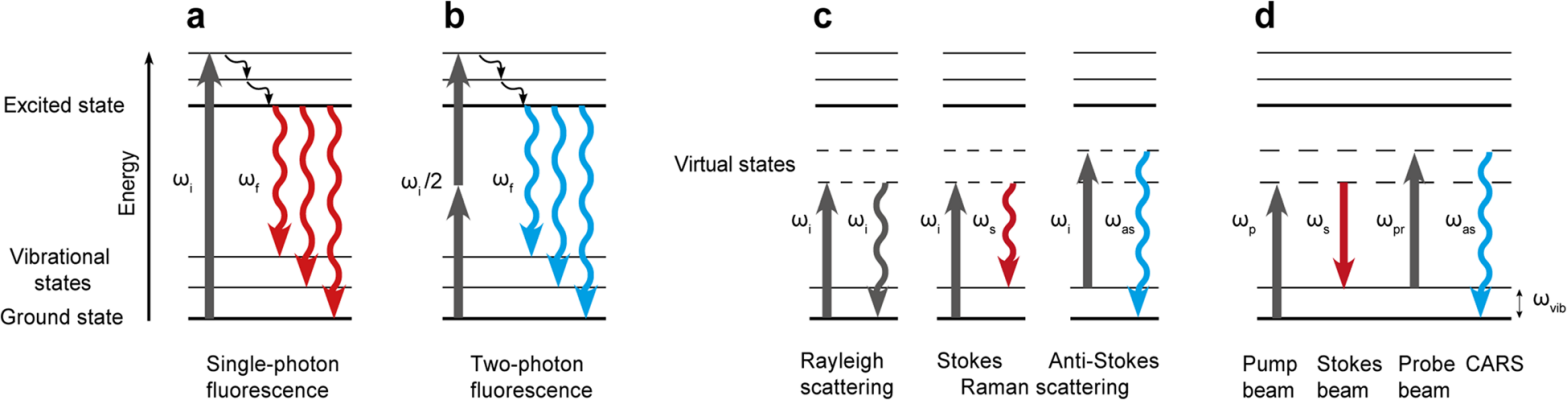
Illustration of single-photon and two-photon fluorescence, Rayleigh and Raman scattering and CARS. Fluorescence (**a** and **b**): Absorption of light at a frequency ω_i_ excites the molecule to a higher electronic energy level. The molecule can revert to the electronic ground state by non-radiative transitions and the emission of fluorescence at frequency ω_f_, which is lower than ω_i_ (red-shifted). In two-photon fluorescence (**b**) the molecule is simultaneously excited by two photons of approximately half the energy necessary for one-photon excitation (ω_i_/2). The resulting two-photon fluorescence signal is blue-shifted compared to the incident light. Light scattering (**c**): Light at a frequency ω_i_ excites a molecule to a virtual state. The molecule can revert to the ground state by elastic light scattering (Rayleigh scattering) or inelastic scattering with an energy loss at frequency ω_s_ (Stokes Raman scattering) or energy gain at frequency ω_as_ (anti-Stokes Raman scattering). CARS (**d**): The CARS process is driven by three photons from at least two different laser sources. A pump beam at frequency ω_p_ excites a molecule from the ground state to a virtual state, which subsequently is depopulated by a Stokes beam at frequency ω_s_. The last photon from the probe beam ω_pr_ excites the molecule to a higher virtual state. In our setup the pump and probe photon are provided by the same laser at ω_p_. The resulting signal at frequency ω_as_ is blue-shifted compared to the incident laser light and – if the conditions are met – coherently amplified [31]

In order to obtain such spatial information optical techniques are frequently utilised for biological issues. Traditional white light optical microscopy gives an overview of sample morphology due to the sample’s intrinsic distinct light transmission. A better differentiated and fine structured image can be obtained by (single-photon) fluorescence microscopy, which, however, for most applications requires labelling of the sample with fluorescent dyes as autofluorescence [24–28] often exhibits either extremely faint or bright, nonspecific signals [29]. In two-photon fluorescence (TPF) microscopy a pulsed laser beam of approximately half of the energy of single-photon excitation is used for excitation (Fig. 2). This method enables a deeper sample penetration and higher resolution in 3D imaging, alongside with reduced photo toxicity [30] but most applications still depend on labelling with dyes, whose toxicity and photo bleaching is often a concern [31].

Label-free and non-targeted techniques have gained importance in recent years, since they avoid such labelling related limitations and allow to study biological samples in a way that more closely reflects their native environment. The use of Raman spectroscopy in microscopy enables label-free and chemically selective imaging (Fig. 2). Molecular identification originates from the specific frequencies of molecular vibrations appearing in a Raman spectrum [31]. A drawback is that spontaneous Raman scattering is a very weak optical effect compared to fluorescence or elastic light scattering. Approximately only one of 10^6^ photons or less of scattered light undergoes an energy loss or gain, so-called Stokes and anti-Stokes Raman scattering (Fig. 2) through interaction with the molecular vibrations [32]. The imaging of a typical biological sample with Raman microscopy is not always suitable as it requires very long image acquisition times, even when intense laser beams are used [31]. In contrast, Coherent anti-Stokes Raman scattering (CARS) is a nonlinear optical technique (for details see Methods section) delivering a Raman equivalent signal of distinctively higher intensity which allows much shorter acquisition times. CARS is therefore highly suitable for imaging applications, especially for the fast, non-destructive imaging of biological samples [31, 33] and has been used e.g. for monitoring the differentiation state of stem cells [34] or the detection of lipids by assessing the strong C-H vibration at a single-band frequency [35].

CARS imaging at different Raman vibrations – so-called hyperspectral CARS imaging (HCARS) – combines imaging with spectroscopy and allows for the differentiation between different substances with the use of sophisticated data evaluation methods [36]: Each pixel in an image consists of a spectrum, which reflects the chemical composition or chemical fingerprint at that position. One possibility to identify single components from such a fingerprint is the spectral decomposition into several constituents – so-called endmembers, each ideally (but not necessarily) representing one substance. The abundance of each endmember in each pixel is subsequently determined by hyperspectral unmixing. The most popular unmixing model (coming from remote sensing) is the linear unmixing model, which assumes that the observed spectrum is a linear combination of endmember spectra with corresponding positive abundances and possibly additive noise [37, 38]. This assumption is not necessarily valid for CARS data, which are intrinsically highly nonlinear due to the signal generation itself. This nonlinearity is conventionally eliminated by using phase retrieval methods (e.g. maximum entropy method [39] or time-domain Kramers–Kronig relations [40]). These methods deliver the imaginary part of the so-called resonant signal, which is directly proportional to the spontaneous Raman signal and thus linear to the analyte concentration. Consequently, a subsequent linear unmixing is feasible. However, phase retrieval is a non-trivial problem, especially when dealing with highly complex biological samples. Accounting for the nonlinearities in the unmixing method itself is an alternative approach. Here we apply a nonlinear unmixing algorithm developed by Heylen et al. [37, 41], which accounts for the nonlinearities in the HCARS data in a geometrical based approach and thus should enable to find suitable endmembers and deliver corresponding abundances without requiring phase retrieval (for details see Methods section).

The aim of this study is to analyse secondary metabolites in glandular *Cannabis* trichomes with distinct spatial resolution. This is achieved by using HCARS imaging in combination with a nonlinear unmixing method. Furthermore, we combine these information with transmission images as well as single-photon and two-photon fluorescence delivering additional morphological information. This multi-modal approach allows to investigate samples regarding their morphology as well as their content of specific secondary metabolites such as THCA and CBDA without extracting the essential oil.

## Results

### Morphology of glandular trichomes

In order to investigate the sample morphology images of glandular *Cannabis* trichomes are recorded with transmission, single-photon fluorescence and scanning electron microscopy (SEM). Fluorescence excitation of glandular trichomes at 561 nm generates two types of fluorescence, a blue-green fluorescence and a red fluorescence. The origin of blue-green fluorescence is probably a mixture of different organic fluorophores. In this paper we refer to it as organic fluorescence. The red fluorescence is probably caused by chlorophyll *a*.

**Fig. 3:**
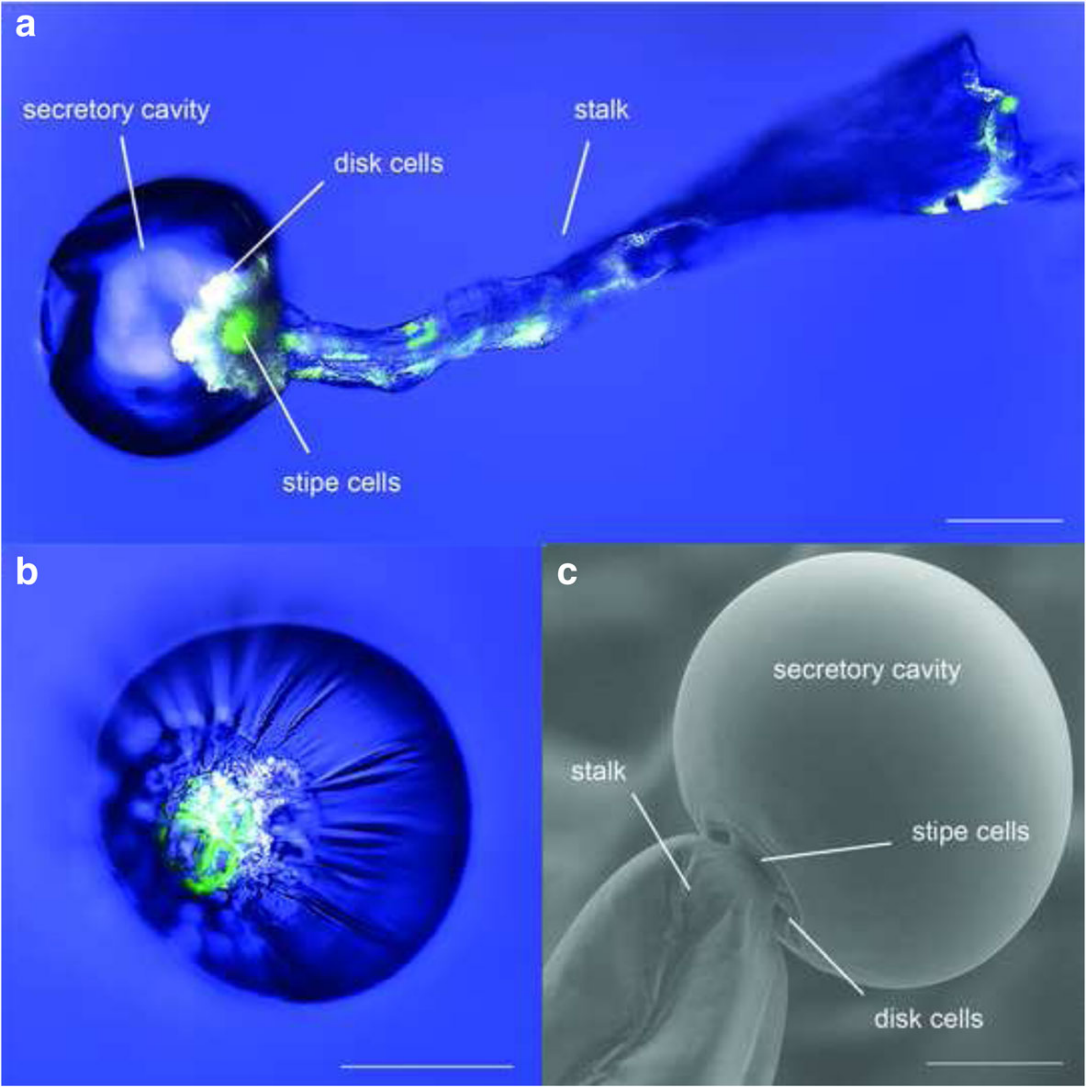
Transmission and single-photon fluorescence images of a glandular trichome (**a**) and of secretory cavity (**b**) of *C. sativa* var. Bedrobinol with 561 nm excitation. Anatomy of glandular trichomes captured with SEM (**c**). Blue: Transmission; White: Fluorescence of organic substances (**em**ission 580–630 nm); Green: Fluorescence of chlorophyll *a* (em 660–700 nm). Scale bars 50 μm (**a** and **b**), 25 μm (**c**)

Combined imaging of these two fluorescence phenomena with transmission (Fig. 3a, b) enables to distinguish between different components of the glandular trichome, which are also resolved by the SEM micrograph (Fig. 3c). The overall structure of the glandular trichome is revealed by the transmission image (Fig. 3a). It consist of a spherical head placed on a stalk. The head divides into a cellular part of strong organic fluorescence, the disk cells, and the secretory cavity. The latter shows a high light transparency rather than fluorescence revealing the presence of essential oil. The stalk shows organic fluorescence and red fluorescence of chlorophyll *a* indicating the localisation of chloroplasts (Fig. 3a). The connection between the stalk and the head is facilitated by so-called stipe cells, which are clearly discriminable by the dominance of the red fluorescence of chlorophyll *a*. The stipe cells are especially well visible in the bottom view of isolated heads without stalks (Fig. 3b). The connection of the stipe cells towards the stalk and secretory cavity is visible in the SEM micrograph (Fig. 3c).

We further investigate the sample morphology by CARS microscopy at a single band frequency and accompanying TPF signals. The CARS lasers (for details see Methods section) are set to probe the Raman vibrational mode at 2861 cm^− 1^, which originates from aliphatic C-H stretching vibrations and enables imaging of aliphatic C-H-rich substances. Additional file 1 shows the results of multi-photon imaging of a secretory cavity of *C. sativa* var. Bedrobinol and *C. sativa* var. Fedora recorded with four detector channels collecting backward (EPI) and forward (F) directed light of shorter and longer wavelength, respectively. In backward direction signals are dominated by TPF (EPI-TPF): Signals of longer wavelength (Additional file 1: Figure S1a and e) show the red fluorescence of chlorophyll *a* in the chloroplasts of stipe and stalk cells and signals of shorter wavelength (Additional file 1: Figure S1b and f) show the organic substance fluorescence inside disk and stalk cells. These observations are in accordance with the single-photon fluorescence phenomena (Fig. 3) with the difference that here TPF allows higher resolution imaging as can best be seen by the more detailed localisation of chloroplasts in the stipe cells (Additional file 1: Figure S1a and e vs. Fig. 3a).

In forward direction signals of longer wavelength (Additional file 1: Figure S1c and g) are mostly emitted by the secretory cavity and attributed to CARS signals (F-CARS): These signals are most likely caused by the aliphatic C-H-rich substances of the essential oil inside the secretory cavity, which would be in accordance with the strong light transparency (Fig. 3a). The forward directed signals of shorter wavelength (Additional file 1: Figure S1d and h) originate from the organic substance fluorescence and are comparable to those observed in backward direction but with much lower intensity. For this reason these signals were not considered further.

As glandular trichomes are often investigated on dried and rehydrated plant material we further investigated the influence of drying by recording time-dependent transmission images during a drying process. Actually, additional morphologic structures occurred during this process (Additional file 2), indicating an artefact formation that probably does not represent the native structure.

### Chemical fingerprints of glandular trichomes

In order to investigate the spatial distribution of metabolites within the glandular trichome we exploit the possibilities of HCARS imaging. We employ nonlinear spectral unmixing with a subsequent hierarchical cluster analysis (HCA) on the data of trichome samples and reference substances to account for chemical fingerprints.

Spectral unmixing of the HCARS data recorded in forward direction (F-HCARS) is achieved by using four endmembers per sample. The chemical fingerprint information is revealed by the endmember spectra and its spatial distribution by the corresponding relative abundance maps. For the drug-type samples two endmembers describe the C-H stretching, one endmember contains fluorescence and the last endmember residual noise. For the fibre-type samples one endmember describes the C-H stretching, one endmember the fluorescence and two endmembers the residual noise. F-HCARS spectra of reference substances are unmixed in the same way but using only two endmembers (CH-stretching, residual noise) due to the reduced sample complexity. Beside the cannabinoids, the most abundant monoterpenes and fatty acids (Fig. 1, [42, 43]) are included in the analysis.

**Fig. 4:**
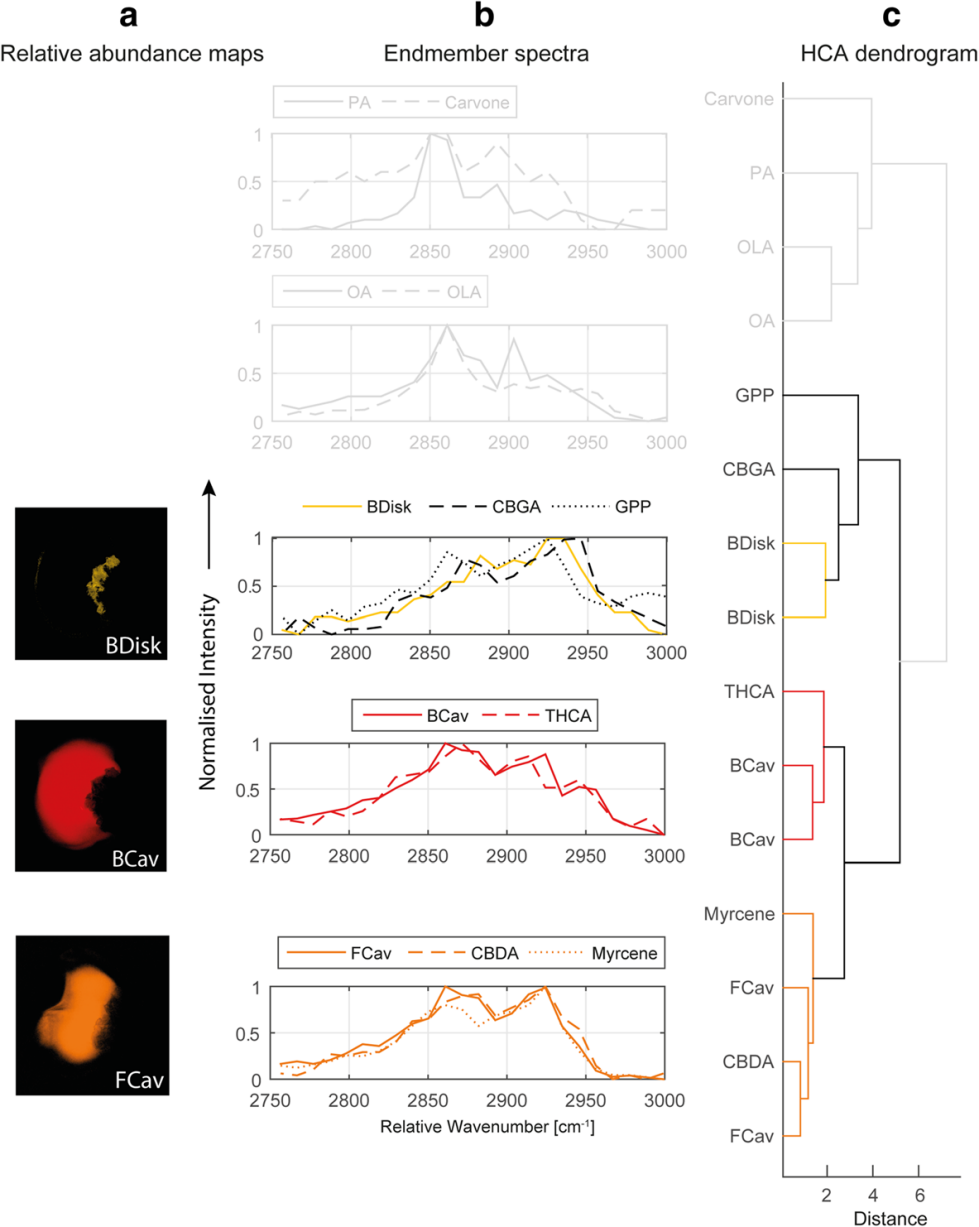
Hierarchical clustering of F-HCARS endmember spectra of reference substances and of glandular trichomes of *C. sativa* var. Bedrobinol (two samples) and *C. sativa* var. Fedora (two samples). Spectral similarity determined by HCA is shown in the dendrogram (**c**), the spectra for reference substances and endmembers are compared (**b**) and the corresponding relative abundance maps (**a**) are presented. Groups connected by coloured lines denote high spectral similarity. Spectra of reference substances, which show no similarity to spectra of glandular trichomes are denoted by grey lines. The data were named according to the sample type (B = Bedrobinol; F = Fedora) and localisation (Cav = secretory cavity; disk = disk cells). Abbreviations according to Fig. 1

Spectral similarities between the endmember spectra of the trichomes and the endmember spectra of reference substances are assessed by HCA. Figure 4 shows the endmember spectra containing the C-H stretching information and corresponding relative abundance maps sorted by the results of the HCA. The dendrogram of the HCA illustrates the spectral similarities.

For drug-type derived trichomes HCA reveals a similarity of the endmembers that show high relative abundances in the secretory cavity (BCav) to the spectrum of pure THCA, indicating a distinct accumulation of THCA inside the secretory cavity. The spectra of the disk cells (Bdisk) are clearly distinguishable from the spectra of the secretory cavity (BCav). They show a similarity to CBGA suggesting an accumulation of this cannabinoid in the disk cells of drug-type derived trichomes. However, the broad C-H signal might also indicate a complex mixture of different C-H-rich substances inside the disk cells.

In contrast to the drug-type derived trichomes endmembers from the secretory cavity of fibre-type derived trichomes (FCav) reveal a spectral fingerprint most similar to CBDA/myrcene according to HCA results. This observation indicates the possibility to differentiate trichomes of the fibre- and drug-type by the spectral fingerprint of the essential oil stored in the secretory cavity, which is not accessible by CARS imaging at single band frequencies.

Unlike disk cells of the drug-type derived trichomes, the disk cells of fibre-type trichomes show no distinct C-H stretching signal significantly different to their surroundings. Therefore, unmixing delivers no endmember exclusively for the disk cells of the fibre-type trichomes and thus no characteristic chemical fingerprint.

The similarity of CBDA and myrcene in the investigated spectral region prevents a distinction between these substances with our method. Consequently, their independent localisation within the samples was not possible. This clearly marks the limitation of the currently applied method. By contrast, THCA shows distinct spectral features, allowing its identification and localisation with the endmember approach.

**Fig. 5:**
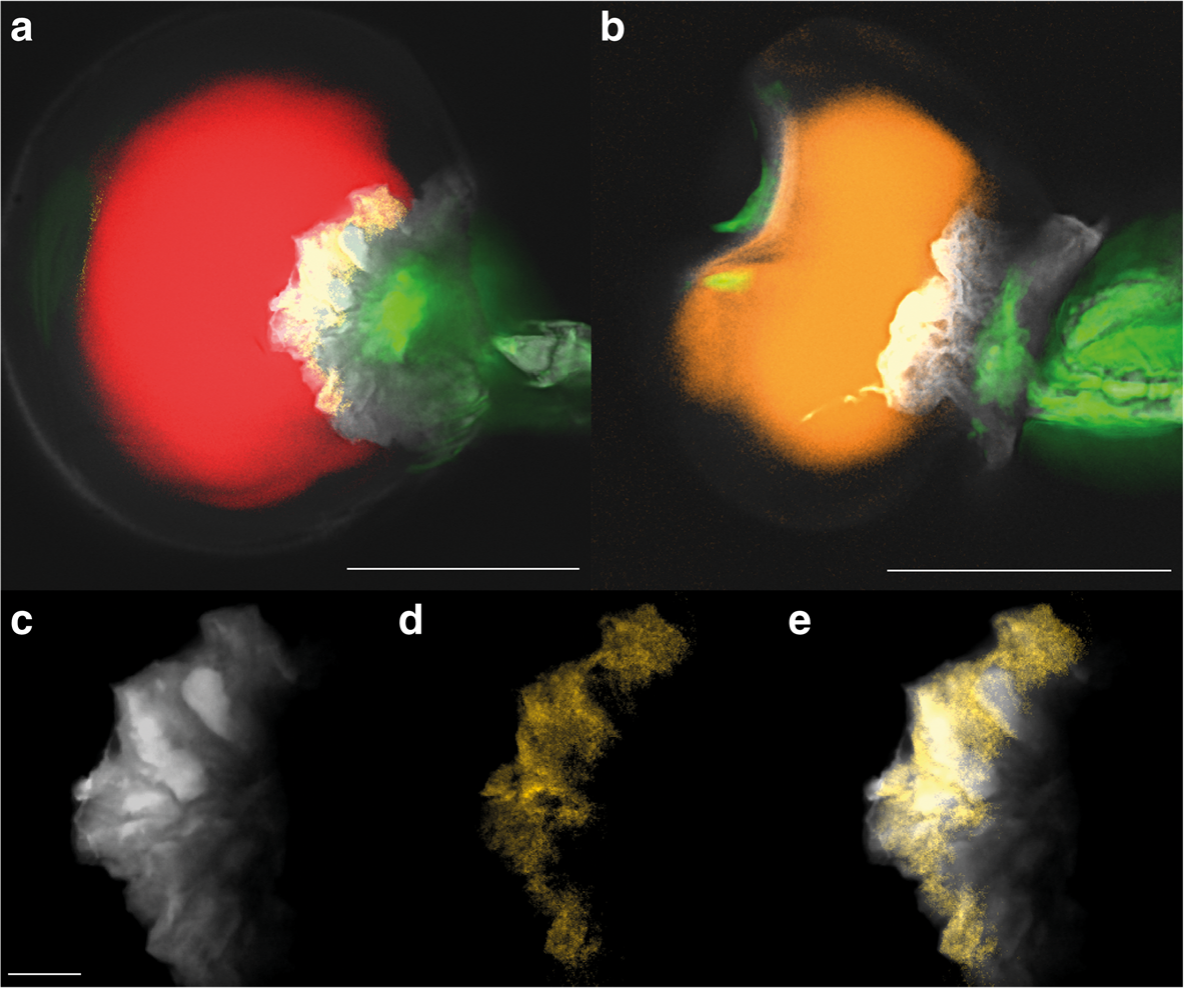
Overlay of F-HCARS relative abundance maps and EPI-HTPF abundance maps of *C. sativa* var. Bedrobinol (**a**) and *C. sativa* var. Fedora (**b**). Red: C-H stretching signal similar to THCA; Yellow: C-H stretching signal most similar to CBGA; Orange: C-H stretching similar to CBDA/myrcene, White: TPF of organic substances (em 380–560 nm); Green: TPF of chlorophyll *a* (em 560–750 nm). Detailed picture of Bedrobinol disk cells (**c**-**e**). TPF of organic substances highlight the disk cell morphology (**c**), F-HCARS signals indicate the presence of CBGA and/or a complex mixture of different aliphatic C-H rich substances (**d**), F-HCARS signals cover the area of organic fluorescence revealing CBGA and/or a complex mixture of different aliphatic C-H-rich substances is almost exclusively localised inside the disk cells (**e**). Scale bars 50 μm (**a** and **b**); Scale bar 10 μm (**c**-**e**)

To provide an entire picture of the spatial distribution of metabolites together with morphological structure, we combine the unmixed F-HCARS data with the corresponding unmixed data recorded in backward direction (Fig. 5), which are dominated by two-photon fluorescence (EPI-HTPF; see also EPI-TPF in Additional file 1). The prominent organic fluorescence of the disk cells in the drug-type trichomes allows to distinguish these cells from the secretory cavity (Fig. 5c). The C-H stretching signal corresponding to CBGA and/or a complex mixture of C-H-rich substances (Fig. 5d) occurs in the same region as the organic fluorescence (Fig. 5e), revealing that the respective compounds are almost exclusively localised inside the disk cells.

### 3D-visualisation of structural and chemical information

Since both CARS and TPF have inherent 3D imaging capabilities [44], we aim to visualise the essential oil distribution and structural differences of high-THCA and high-CBDA containing trichomes. Therefore, the intensity of the C-H stretching band at 2907 cm^− 1^ of drug- and fibre-type samples in different z planes was measured and subsequently merged to a 3D image. Simultaneously registered EPI-signals deliver further structural information by TPF of organic substances (em 380–560 nm) and of chlorophyll *a* (em 560–750 nm).

The 3D animations of a drug-type (Additional file 3) and a fibre- type glandular trichome (Additional file 4) show that the C-H stretching signal of the essential oil (red and orange, respectively) is only dominant in the secretory cavities. The stalk is dominated by TPF of organic substances (grey). Localisation of chloroplasts is revealed by TPF of chlorophyll *a* (green). The secretory cavity of the fibre-type trichome shows a less prominent C-H stretching signal compared to the drug-type trichome and is more influenced by TPF from cell walls. It is worth noting that fibre-type trichomes appear to have smaller spatial dimensions compared to drug-type trichomes regarding the total length (260 μm for fibre-type versus 400 μm for drug type) and the diameter of the secretory cavity (70 μm versus 80 μm).The 3D structure of a dissected glandular head from a drug-type trichome sheds light at the connection between the stipe cells, disk cells and the secretory cavity (Additional file 5). Differentiation between the disk cells and secretory cavity is realized by the pronounced TPF of organic substances (grey) from the disk cells compared the low fluorescence signals from the secretory cavity. The stipe cells are discriminable by TPF of chlorophyll *a* in backward direction (green, em 560–750 nm), which can also be seen in forward direction (red). F-HCARS data suggest that the forward directed light in the CARS channel (em 560–750 nm) is only due to fluorescence, since it shows a slightly increasing baseline but does not contain C-H stretching signals. Since the intensity in the F-CARS channel coincides with the position of the stipe cells, we assume it to be TPF signals of chlorophyll *a*.

## Discussion

Chemical fingerprinting of secondary metabolites in biological samples requires a high spectral selectivity and sophisticated data evaluation. In complex biological material this is even more complicated due to the autofluorescence of the sample matrix, which however can also be used to get a more complete picture of the sample. In the here investigated glandular trichomes of a drug- and fibre-type of *C. sativa* excitation with 561 nm results in the plant-typical blue-green fluorescence and an additional (far-)red fluorescence, which is known to be caused by chlorophyll *a* [24–27]. The blue-green fluorescence is probably primarily the result of highly fluorescent substances in the cell wall (e.g. ferulic acid) [25]. In a study by Talamond et al. [24] it has been shown that hyperspectral imaging of the blue-green fluorescence band with subsequent multivariate spectra separation in principle enables imaging of single phenolic metabolites in plant material (e.g. caffeine in coffee leaves).

However, cannabinoid acids show pronounced fluorescence only at UV excitation [21], while we applied VIS excitation. Furthermore due to their structural similarity they have very similar fluorescence spectra thus preventing a clear differentiation [21]. These properties prevent a selective chemical identification of the essential oil in glandular trichomes even with sophisticated unmixing methods just based on fluorescence. Therefore, we use the blue-green fluorescence only to obtain a general distribution of organic fluorophores in the glandular trichomes. This organic fluorescence enables in combination with the fluorescence of chlorophyll *a* and transmission images the clear differentiation between cellular components (stalk, stipe and disk cells) and non-cellular components (secretory cavity) [10, 45, 46].

For the localisation of secondary metabolites in the glandular trichomes HCARS imaging is used, which contrary to the broad fluorescence bands delivers more and sharper peaks, enabling a more selective chemical identification. Garbacik et al. [47] already showed that HCARS imaging in the C-H stretching region is applicable for the detection of THCA inside the glandular trichome of dried and rehydrated plant material, but neither a clear spatial localisation was possible nor were further metabolites investigated. We obtain a more detailed view of the glandular trichomes both in terms of morphology and chemical information with HCARS imaging first by using a structure preserving sampling strategy and second by introduction of a nonlinear unmixing method for data analysis.

Regarding the first point we use intact fresh trichomes carefully bursted from *Cannabis* flowers frozen in liquid nitrogen. Our measurements suggest that imaging of dried trichomes not necessarily reflects their native state since we were able to show that pseudo-morphological structures appear upon drying. Nevertheless, we cannot exclude that the cryofixation of the trichomes performed in this study might lead to the formation of cryo-artefacts, e.g. the damage of cell organelles or subcellular structures like the secretory vesicles of the cavity [45]. However, this would not affect our current conclusions.

Regarding the second point we use an unmixing model originally employed in the field of geosensing that geometrically takes into account the nonlinear nature of the HCARS data and thus presumedly better reflects the actual spectral variations and abundances of the data. This approach captures fine spectral differences of the C-H stretching bands and separates them from the fluorescence and residual noise background. It has to be emphasised that such a geometry-based method does not necessarily provide access to the real concentration of an analyte but rather reflects the spectral differences in an image. For this reason we use terms from the field of unmixing (e.g. endmember, abundance) instead of more chemical related terms (e.g. concentration). To our knowledge, this is the first time that such a nonlinear unmixing method is applied to evaluate nonlinear CARS data of biological samples. Previous studies in the field of Raman micro-spectroscopy used linear mixing models like the popular VCA algorithm [48–50], which was even applied to CARS data [51].

Unmixing of the raw F-HCARS data accounts mostly for variations of the absolute intensity in the hyperspectral images, yielding a height-like profile showing a downward gradient from the inner part of the secretory cavity to the outer part. Therefore, in order to visualise spectral differences of the secretory cavity and the disk cells of the drug type trichomes, the F-HCARS data are normalised before unmixing. Otherwise, unmixing can be performed on the non-normalised raw data as a means of noise reduction, e.g. on the corresponding EPI-HTPF data.

Our results suggest that the new approach is suitable for chemical fingerprint imaging. In contrast to an earlier publication only one of the two reported CARS spectra (THC1) is detected [47]. The unique spectrum of THCA enables its spectral identification in the trichomes. The combined results of HCA and abundance maps reveal that the secretory cavities of the drug-type derived trichomes contain mostly THCA. However, the similarity of spectra acquired from the secretory cavity of fibre-type samples or from the disk cells of drug-type trichomes to spectra of reference compounds is less clear. Spectra from the secretory cavities of the fibre-type derived trichomes fit well to spectra from both, CBDA and myrcene. As a consequence we cannot localise these substances individually in the samples. Likewise, spectra from disk cells of the drug type are similar to CBGA spectra, but show also slight differences, which may indicate the presence of a complex mixture of different aliphatic C-H rich substances instead. Nevertheless, the fact that we found THCA and CBDA/myrcene in the secretory cavity of the drug- and fibre-type, respectively, is in accordance with the chemo-type [20, 52–54]: While the drug-type mostly accumulates THCA, it is known that the fibre-type accumulates CBDA as main cannabinoid inside the secretory cavity. Moreover, the spatial determination of the cannabinoids in our experiments of the drug-type is in accordance with literature-known investigations regarding the biosynthesis: While CBGA is thought to be produced inside the disk cells, the synthesis of THCA is most likely located inside the secretory cavity [55]. It is worth noting that by reason of convergent evolution, it is not possible to apply our technique to discriminate between different members of the *Cannabis* genus since it considers only the chemotype [55–57].

Beside the lacking distinction of some chemically related compounds (e.g. CBDA and myrcene) one of our main drawbacks currently is the low sensitivity hindering the detection of compounds of lower abundance. The strong non-resonant background and the nonlinear character of CARS allow only the imaging and chemical identification of the most abundant substances [58]. Therefore, we currently cannot provide a more detailed picture of the localisation of the various different secondary metabolites.

To overcome these limitations a combination of HCARS imaging with subsequent Raman micro-spectroscopy on regions of interest (e.g. secretory cavity, disk or stipe cells) might improve chemical selectivity and even allow the detection of less abundant components. In contrast to HCARS, Raman micro-spectroscopy gives easy access to the more discriminative fingerprint region below 2000 cm^− 1^. Long acquisition times during Raman measurement would be kept to a minimum as Raman would only be used to measure a specific region of interest and not the whole sample. As spontaneous Raman is a linear effect it shows increased sensitivity towards lower abundant substances compared to CARS and might even allow absolute quantification for compounds of interest.

## Conclusion

In the work presented we successfully apply a CARS imaging setup to visualise the main cannabinoids in intact *Cannabis* glandular trichomes and to distinguish between different cell types. We also employ absorption and (two-photon) fluorescence spectroscopy to obtain more structural information in order to identify different compartments and cells types. HCARS imaging in combination with a nonlinear spectral unmixing method offers the possibility to discriminate between THCA-rich and CBDA-rich *Cannabis* plants with a label-free method on single-trichome level with chemical selectivity and high spatial resolution. Furthermore, the essential oil distribution and the whole trichome morphology can be visualised in 3D by a combination of CARS and TPF. Since this kind of visualisation allows to screen for differences in the chemical composition of single trichomes, the variance within a single plant or among the same genotype is now accessible. Additionally, this method can be used to investigate the influence of abiotic factors -like temperature and light- on the essential oil composition, or to determine optimal harvest times to optimize production conditions.

## Methods

### Reagents

Δ^9^-tetrahydrocannabinolic acid (THCA) and cannabidiolic acid (CBDA) were purchased from THC Pharm (Frankfurt am Main, Germany). Cannabigerolic acid (CBGA) was obtained from Taros Chemicals (Dortmund, Germany). Olivetolic acid (OA) was purchased from Santa Cruz Biotechnology, Inc. (Heidelberg, Germany). Geranyl diphosphate (GPP) was synthesised according to Woodside et al. [59]. Carvone, myrcene, palmitic acid (PA) and oleic acid (OLA) were purchased from Sigma Aldrich (Darmstadt, Germany).

### *C. sativa* samples

*C. sativa* var. Fedora seeds (Botanik Sämereien GmbH, Wädenswil, Switzerland) were germinated on wet tissue paper, transferred to hydrocorrels (Plagron, Netherlands) and cultivated in a climate chamber (CLF PlantMaster, CLF Plant Climatics GmbH, Germany) under long-day conditions (18 h light / 6 h dark) at 25 °C and 110 μmol m^− 2^ s^− 1^ irradiation intensity. Seedlings were watered with FloraGro, FloraMicro and FloraBloom (0.03% each, General Hydroponics Europe). Three to 4 weeks after germination nutrition solution concentrations were changed to 0.07% each. For the initiation of flowering light conditions were set to a 12 h light / 12 h dark cycle, the light intensity was increased to 150 μmol m^− 2^ s^− 1^ and nutrition solutions were changed to 0.15% FloraGro, 0.1% FloraMicro and 0.05% FloraBloom.

*C. sativa* var. Bedrobinol plants were supplied by Bedrocan BV (Veendam, Netherlands).

### Trichome isolation

Isolation of single fresh trichomes of *C. sativa* var. Bedrobinol and *C. sativa* var. Fedora flower buds was performed from plant material snap frozen in liquid nitrogen. Frozen trichomes were broken from the sepal surface directly over a coverslip (Cover slips # 1, Menzel-Gläser) either with a cooled stainless steel tip or with a lasso formed by a tungsten wire. The isolation was performed under a stereo zoom microscope (Zeiss, Jena, Germany) at room temperature.

### Optical setup

All imaging was conducted with a modified Leica TCS SP8 CARS (Leica Microsystems CMS GmbH).

Coherent anti-Stokes Raman scattering (CARS) generates a coherently driven transition [44] with a nonlinear signal that is in an approximately quadratic relation to the analyte concentration and usually is several magnitudes higher than the corresponding spontaneous Raman signal [60]. The combined action of a pump laser beam at a frequency ω_p_ and a Stokes laser beam at a frequency ω_s_ generates a coherent superposition of the ground state and the first excited vibrational state at the difference frequency ω_p_ *-* ω_s_ (Fig. 2) [31]. Through interaction with the probe beam (here identical to the pump beam), the vibrational coherence is converted into a detectable signal at frequency 2ω_p_ *-* ω_s_.

For (H)CARS imaging a picosecond one-box laser system (picoEMERALD™, APE GmbH) was used. Hyperspectral images were recorded by tuning the pump laser frequency from 787.5 nm to 839.9 nm (step size 0.7 nm, 76 steps, scan speed 10 Hz, 1024 × 1024 pixel) while keeping the Stokes laser constant at 1064 nm corresponding to a spectral region of 2500 cm^− 1^ – 3300 cm^− 1^. CARS and two-photon fluorescence signals were collected with an IR-light optimised HC PL IRAPO 40x/1.10 WATER objective and split through optical windows into light of longer and shorter wavelength (560–750 nm and 380–560 nm, respectively) both in forward and backward direction. This resulted in four simultaneously acquired channels using photomultiplier tubes (PMTs) as detectors: F-CARS, F-TPF and two EPI-TPFs. Hyperspectral data are denoted with and additional H (e.g. F-HCARS).

Single-photon fluorescence images were obtained at the same instrument but in a confocal setup with 561 nm excitation wavelength (DPSS Laser), prism spectrum generation and spectrum separation by cascade-like movable mirrors and recording in backward direction with two HyD detectors set to 580–630 nm and 660–700 nm. Specifications of the HyD detectors can be found in the description by the manufacturer [61]. Transmission images were recorded with PMTs in forward position (F-CARS and F-TPF). In order to determine the origin of the fluorescence we also recorded fluorescence emission spectra by collecting light stepwise at different emission bands (em 570–745 nm, step size 5 nm, 35 steps).

### Image processing

All data processing was performed using Matlab version R2015a.

In unmixing of hyperspectral images, one usually assumes that the endmembers and abundances are unknown [37]. In a first step the endmembers have to be identified by an endmember extraction algorithm (EEA) [37]. Several of these EEAs assume that endmember spectra are present in the data itself. These so-called purest pixels contain only a single endmember component. In a second step the abundance of each endmember is usually estimated by minimisation of the error by a non-negative least squares algorithm.

We used a nonlinear unmixing chain developed by Heylen et al. [37, 41], where endmember extraction and determination of the corresponding abundances is based on graph-geodesic distances between the pixels located in the n dimensional spectral space. The distance metric is based on internal distances of the pixel spectra along a K-nearest neighbour graph and thus accounts for nonlinearities in the data cloud [41]. Applying this method to HCARS data is based on the following idea: The spectral differences of the pixel in a hyperspectral image reflect the underlying nonlinear CARS process, namely differences of the quadratically concentration dependent resonant signals and the nonresonant background. Thus, the geometrical representation of the pixel spectra in n-dimensional spectral space should yield a nonlinear shape of the resulting data cloud [41]. An unmixing model considering spectral differences along a K-nearest neighbour graph should therefore better reflect the nonlinear nature of CARS than a linear unmixing model, which does not account for the internal structure of the data cloud.

After construction of the K-nearest neighbour graph and calculation of the geodesic distances on this graph a distance geometric version of the maximum distance algorithm (DMaxD) extracts the endmember spectra by using the graph-geodesic distances [37]. The DMaxD algorithm first assumes that the pixels with smallest and largest magnitude are endmembers. Further endmembers are added iteratively by orthogonal projection of the remaining pixels on the hyperplane through the already retrieved endmembers. The pixel with the largest distance to this hyperplane is selected as new endmember [37]. Abundance maps of the retrieved endmembers are calculated by a distance simplex projection unmixing (DSPU) algorithm, which is a distance geometric version of the simplex projection unmixing algorithm (SPU) and also uses the graph-geodesic distances as distance metric [37, 41, 62]. The algorithm makes use of the fact that a constrained least squares problem is geometrically equivalent to a projection operation on a simplex.

In difference to Heylens approach we calculated the K-nearest-neighbour graph by the KD-tree method instead of using the suggested GPU parallelisable algorithm [37]. The connectivity parameter K was set to 10.

Unmixing of CARS signals was applied in the range of the C-H stretching region of the F-HCARS images (2760–3000 cm^− 1^). Laser intensity fluctuations along x and y were eliminated by smooth two-dimensional median filtering along a 3-by-3 neighbourhood around the corresponding pixel. To force an unmixing regarding the spectral shape instead of spectral intensity, the data were vector normalised prior to umixing. The number of endmembers was chosen so that at least one endmember contained mostly spectral noise. This resulted in four endmembers for trichome samples and two for reference substances.

Normalised endmember F-HCARS spectra were compared to each other by a hierarchical cluster analysis (ward agglomerative algorithm [63]).

TPF emission images at 380–560 nm and at 560–750 nm were obtained by unmixing of the two EPI-HTPF data in the range from 787.5 nm to 839.9 nm pump excitation. We used two endmembers, one endmember mostly describing the increasing baseline typical for fluorescence and one endmember describing residual noise. Normalisation on these data was not applied, since this would render fluorescence indistinguishable from the background noise: Fluorescence information here is mostly contained in the spectral intensity rather than spectral shape. Normalisation would therefore erase this spectral information.

### Scanning electron microscope images

Fresh *C. sativa* flower buds were directly mounted on specimen holders and scanning electron microscope (SEM) images were immediately recorded on a Quanta 200 F (FEI, OR, USA) under low vacuum mode (130 Pa).

## Additional files

Additional file 1: CARS and two-photon fluorescence (TPF) images in backward and forward direction of secretory cavity of *C. sativa* var. Bedrobinol (a-d) and *C. sativa* var. Fedora (e-h) at pump 812.6 nm and Stokes 1064 nm (2861 cm^− 1^). Green: TPF of chlorophyll *a* in backward direction (em 560–750 nm) (a, e); White: TPF of organic substances in backward direction (em 380–560 nm) (b, f); Magenta: CARS signal of the essential oil in forward direction (c, g); White: TPF of organic substances in forward direction (d, h). Scale bars 50 μm. (TIF 20253 kb)

Additional file 2: Time dependent degradation of a glandular trichome of *C. sativa* var. Bedrobinol. Fresh trichome (a) after 1 day at 27 °C (b) after 2 days at 27 °C (c). Scale bar 50 μm. (TIF 3328 kb)

Additional file 3: Overlay of a CARS- and two-photon fluorescence (TPF) 3D image of a glandular trichome of *C. sativa* var. Bedrobinol. Red: CARS signal at 2872 cm^− 1^ in forward direction; gray: TPF of organic substances in backward direction (em 380–560 nm); green: TPF of chlorophyll *a* in backward direction (em 560–750 nm). The colour code was chosen in accordance to the observations made by hyperspectral CARS imaging. (AVI 1085 kb)

Additional file 4: Overlay of a CARS- and two-photon fluorescence (TPF) 3D image of a glandular trichome of *C. sativa* var. Fedora. Orange: CARS signal at 2907 cm^− 1^ in forward direction; gray: TPF of organic substances in backward direction (em 380–560 nm); green: TPF of chlorophyll *a* in backward direction (em 560–750 nm). The colour code was chosen in accordance to the observations made by hyperspectral CARS imaging. (AVI 845 kb)

Additional file 5: Overlay of a CARS- and two-photon fluorescence (TPF) 3D image of secretory cavity and disk cells of *C. sativa* var. Bedrobinol. Red: CARS signal at 2907 cm^− 1^ in forward direction; gray: TPF of organic substances in backward direction (em 380–560 nm); green: TPF of chlorophyll *a* in backward direction (em 560–750 nm).The colour code was chosen in accordance to the observations made by hyperspectral CARS imaging. (AVI 1751 kb)

## Abbreviations

B: Bedrobinol
CARS: Coherent anti-Stokes Raman scattering
Cav: Secretory cavity
CBD: Cannabidiol
CBDA: Cannabidiolic acid
CBGA: Cannabigerolic acid
Disk: Disk cells
DMaxD: Maximum distance algorithm
DSPU: Distance simplex projection unmixing algorithm
EEA: Endmember extraction algorithm
em: Emission
EPI-CARS/-TPF: Backward directed light of CARS/TPF
F: Fedora
F-CARS/-TPF: Forward directed light of CARS/TPF
GC/MS: Gas chromatography–mass spectrometry
GPP: Geranyl diphosphate
HCA: Hierarchical cluster analysis
HCARS: Hyperspectral coherent anti-Stokes Raman scattering
HTPF: Hyperspectral two-photon fluorescence
LC/MS: Liquid chromatography–mass spectrometry
NMR: Nuclear magnetic resonance
OA: Olivetolic acid
OLA: Oleic acid
PA: Palmitic acid
PMT: Photomultiplier tube
SEM: Scanning electron microscopy
SPU: Simplex projection unmixing algorithm
THC: Δ^9^-Tetrahydrocannabinol
THCA: Δ^9^-Tetrahydrocannabinolic acid
TPF: Two-photon fluorescence
VCA: Vertex component analysis
