# Supplementary figures and images for: Chemical fingerprinting of single glandular trichomes of Cannabis sativa by Coherent anti-Stokes Raman scattering (CARS) microscopy
Source: BMC Plant Biol. 2018 Nov 12;18:275. doi: 10.1186/s12870-018-1481-4 (PMC6233497; doi:10.1186/s12870-018-1481-4)

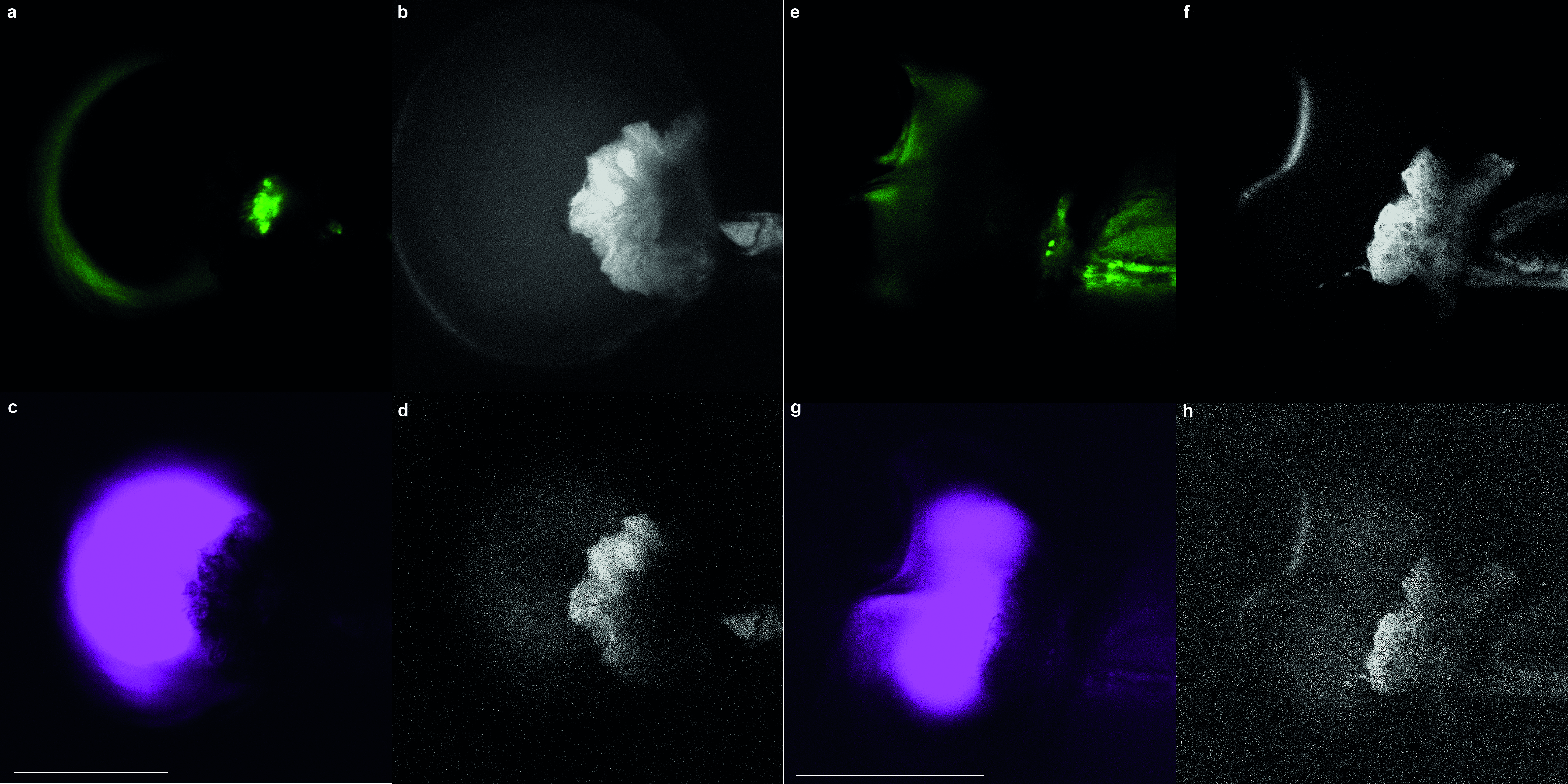

Supplement: Supplementary file 1 — CARS and two-photon fluorescence (TPF) images in backward and forward direction of secretory cavity of C. sativa var. Bedrobinol (a-d) and C. sativa var. Fedora (e-h) at pump 812.6 nm and Stokes 1064 nm (2861 cm− 1). Green: TPF of chlorophyll a in backward direction (em 560–750 nm) (a, e); White: TPF of organic substances in backward direction (em 380–560 nm) (b, f); Magenta: CARS signal of the essential oil in forward direction (c, g); White: TPF of organic substances in forward direction (d, h). Scale bars 50 μm. (TIF 20253 kb) [file 12870_2018_1481_MOESM1_ESM.tif]

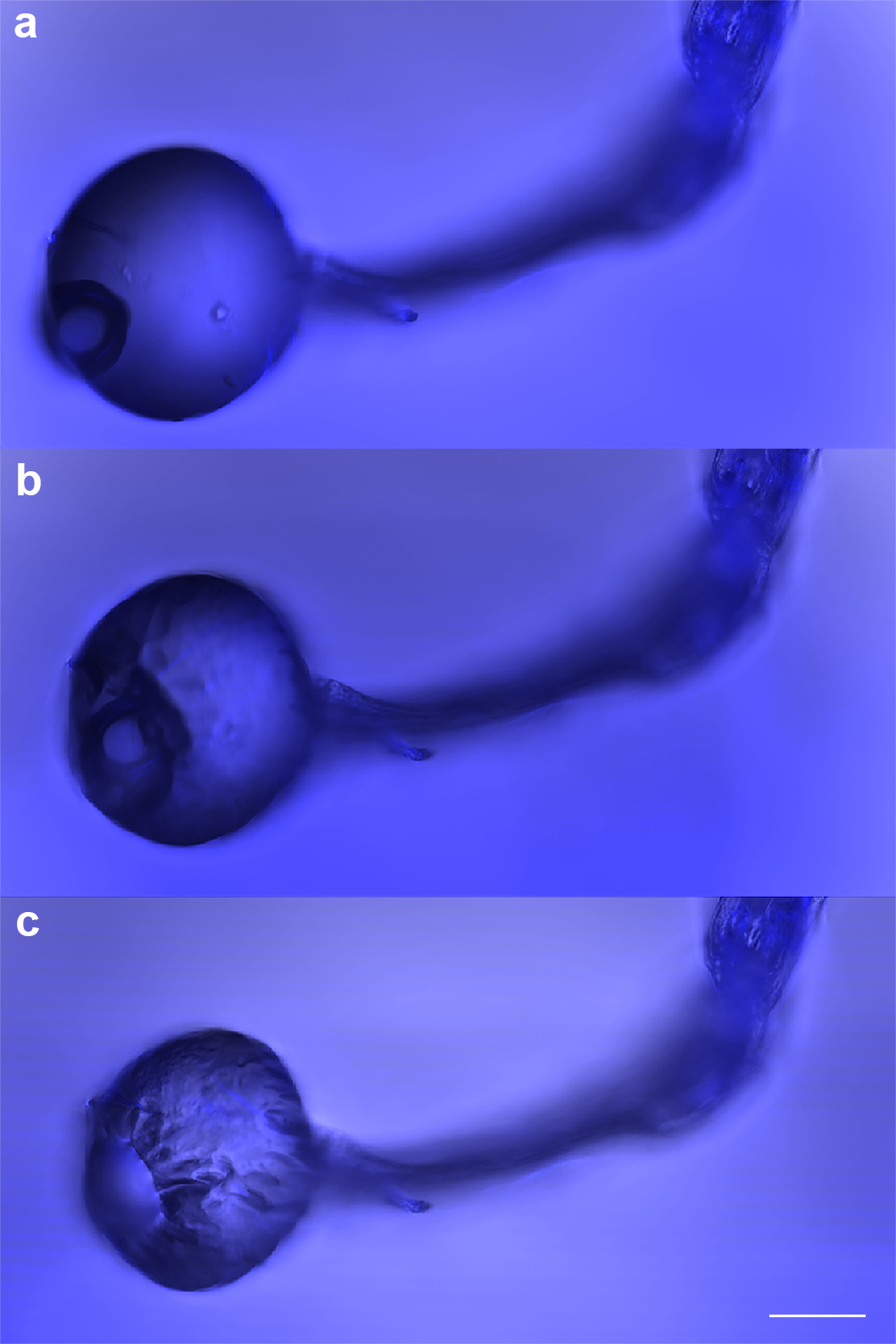

Supplement: Supplementary file 2 — Time dependent degradation of a glandular trichome of C. sativa var. Bedrobinol. Fresh trichome (a) after 1 day at 27 °C (b) after 2 days at 27 °C (c). Scale bar 50 μm. (TIF 3328 kb) [file 12870_2018_1481_MOESM2_ESM.tif]
